# Detecting and Quantifying Topography in Neural Maps

**DOI:** 10.1371/journal.pone.0087178

**Published:** 2014-02-05

**Authors:** Stuart Yarrow, Khaleel A. Razak, Aaron R. Seitz, Peggy Seriès

**Affiliations:** 1 Institute for Adaptive and Neural Computation, School of Informatics, University of Edinburgh, Edinburgh, United Kingdom; 2 Department of Psychology, University of California, Riverside, California, United States of America; Rutgers University, United States of America

## Abstract

Topographic maps are an often-encountered feature in the brains of many species, yet there are no standard, objective procedures for quantifying topography. Topographic maps are typically identified and described subjectively, but in cases where the scale of the map is close to the resolution limit of the measurement technique, identifying the presence of a topographic map can be a challenging subjective task. In such cases, an objective topography detection test would be advantageous. To address these issues, we assessed seven measures (Pearson distance correlation, Spearman distance correlation, Zrehen's measure, topographic product, topological correlation, path length and wiring length) by quantifying topography in three classes of cortical map model: linear, orientation-like, and clusters. We found that all but one of these measures were effective at detecting statistically significant topography even in weakly-ordered maps, based on simulated noisy measurements of neuronal selectivity and sparse sampling of the maps. We demonstrate the practical applicability of these measures by using them to examine the arrangement of spatial cue selectivity in pallid bat A1. This analysis shows that significantly topographic arrangements of interaural intensity difference and azimuth selectivity exist at the scale of individual binaural clusters.

## Introduction

Topographic neural maps of the body or features of the sensory environment are a near-ubiquitous phenomenon in the brains of many species. Topographic maps are found in the visual [1], auditory [2], somatosensory [3] and motor [4] areas, in many subcortical structures, and even in areas of cortex associated with higher functions [5]. The widespread occurrence of topographic maps, and the fact that they are in many cases conserved through multiple stages of neural processing, strongly suggest that they are associated with some significant evolutionary advantage.

Many experimental techniques have been used to observe topographic maps, including single-electrode [1] and multi-electrode [6] electrophysiology, optical intrinsic signal imaging [7], fMRI [8], calcium imaging [9] and microstimulation [3]. Topography of intrinsic neuronal properties has also been observed using intracellular recording techniques (see [10] for a review). These methods vary in spatial resolution, and in the number of points (neurons or assemblies of neurons) at which response properties can be measured simultaneously.

**Figure 1:**
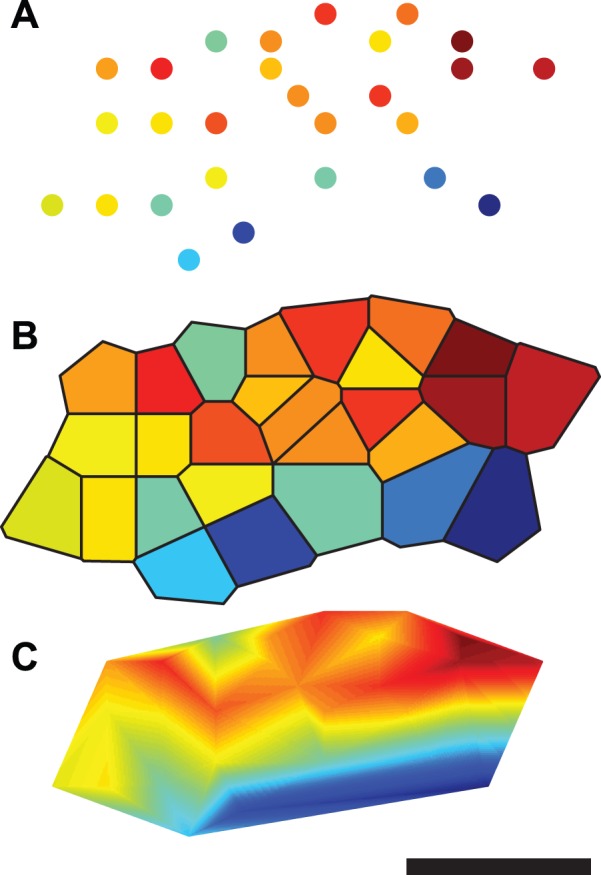
Three visualizations of the same sparsely sampled cortical map data. The perceived orderliness of a topographic map can vary depending on how the data is presented. This figure shows identical mapping data (an azimuth map in pallid bat A1) plotted in three ways: (A) no interpolation, (B) Voronoi tessellation (nearest neighbor interpolation), and (C) linear interpolation. Color indicates the value of the mapped tuning parameter and color scaling is continuous and consistent across panels. Scale bar is approximately 0.5 mm. Analysis results for this map (measure values and Benjamini-Hochberg corrected 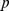-values; see Results): 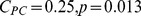; 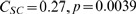; 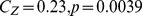; 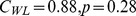; 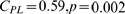; 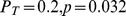; 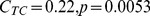.

Intuitively, the defining property of a topographic map is that, within the map, anatomically proximate locations are occupied by neurons with similar functional properties. However, distilling this intuitive understanding into a more rigorous definition of topography is not straightforward [11]. Any formal definition of topography rests upon how similarity of both functional properties and anatomical location are quantified; different methods of measuring difference or distance lead to different definitions of topography. Concordant with the prevalence of a loosely-defined notion of topography, neural topographic maps are normally identified subjectively and described qualitatively, and surprisingly few attempts have been made to quantify the degree of topography in experimentally-observed maps. Whilst the subjective and qualitative treatment of neural maps has well-established utility, some situations demand a more rigorous approach. Topography on a scale close to the resolution limit of the observation technique may be difficult to identify, as the spatial density of measurements required to characterize the map becomes difficult to achieve. Also, map measurement techniques that rely on serial measurements (e.g. single-electrode electrophysiology) limit the number of points that can be measured in any one experimental subject. In cases such as these, we suggest that detecting the presence of a topographic map is a non-trivial task. It is also difficult to reliably estimate the degree of topographic organization by visual inspection [12], and the method used to visualize the map can affect the perceived degree of topography (see Figure 1; the method used in panel A might be considered advantageous as its lack of interpolation avoids any implicit assumptions about the properties of neurons between the measured locations). An objective method of detecting significant order would be advantageous, as it would eliminate the need to rely upon subjective judgement. Topographic maps are known to be sensitive to both biological and environmental factors and a well-understood quantitative measure of topography would be of broad utility for making objective comparisons between maps.

Measures that quantify the local consistency of neuronal selectivity have been used to analyze experimental data; for example the Local Homogeneity Index [13], and the Local Coherence Index [14]. These methods assign high scores where neighboring cells are similarly tuned, but they do not quantify topography in the sense of a broader preservation of neighborhood relations. Kaschube and collaborators have published several quantitative analyses of maps in primary visual cortex (V1), using quantities such as ocular dominance column spacing [15] and orientation pinwheel density [16], but again these measures do not quantify topography as such. Polley et al. [17] and Bandyopadhyay et al. [18] illustrated tonotopic maps by producing scatter plots of characteristic frequency against position on the rostrocaudal axis of the primary auditory cortex (A1), and this approach was extended by Zheng [19], who quantified maps by computing correlation coefficients (also between frequency and position on the rostrocaudal axis of A1). Zheng [19] also computed average pairwise distances between 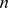
th-nearest neighbor cells with matching characteristic frequencies and used this measure in a bootstrap analysis to demonstrate statistical significance. Guo et al. [20] used a vector averaging approach to make spatially resolved estimates of tonotopic map precision in several regions within the mouse auditory cortex, and used nonparametric statistical tests to compare tonotopy across regions and a number of different experimental conditions. Alvarez et al. [12] defined measures of topographic organization and lateral asymmetry for retinotopic maps, but these were based on differences from a predefined reference pattern; this is only a viable approach if such an ideal map can be defined. In cases where the dimensionality of the map matches the dimensionality of the space that it represents (such as in a retinotopic map) it is trivial to define an ideal mapping, but this is not the case where there is a difference in dimensionality between map and feature spaces – there is no unique ideal map. Willshaw [21] measured the emergence of topography in a model of retinocollicular map development by quantifying receptive field size and overlap, making use of the fact that in a mature, ordered map, receptive fields tend to be local and less-overlapping. Willshaw et al. also went on to quantify topography in one-to-one retinocollicular maps by computing the size of the largest map subdomain within which neighborhood relations were perfectly preserved [22]. However, similarly to the approach of Alvarez et al., this method depends on the existence of a well-defined ideal mapping.

The literature on iterative map generation methods, such as self-organizing feature maps, contains a wealth of information on quantifying the ‘goodness’ of topographic mappings (for reviews, see [11], [23], [24]) and much of this is applicable to biological maps. In this article, we examine seven map measures drawn from the map development modeling literature (Pearson distance correlation, Spearman distance correlation, Zrehen's measure, topographic product, topological correlation, wiring length and path length), with the aim of establishing an objective, quantitative method for comparing experimentally characterized maps and detecting statistically significant topography. We first assess the statistical power of each measure when applied to the detection of different types of map: linear gradient; convoluted, similar to maps of orientation in V1; and maps composed of randomly arranged homogeneous clusters. Based on the results of these simulations, we found that six of the measures were well suited to detecting topography and only one (wiring length) was less useful due to low statistical power.

We then go on to illustrate the use of map measures to detect topography in experimental data. The recently identified systematic arrangement of azimuth selectivity [25] and corresponding binaural selectivity (interaural intensity difference [26]) in A1 of the pallid bat (*Antrozous pallidus*) is an example of a very small map that has been identified using single unit recordings. Because the systematic representation covers only a small area of cortex (<3mm^2^) and because of the limited time available for making serial single-neuron recordings in each animal, Razak [25] identified the systematic map based on relatively few recordings (between 14 and 36 cells per animal). Here we quantify the topography in characteristic frequency, source azimuth and interaural intensity difference (IID) selectivity in A1 of the pallid bat. In addition to the well-known tonotopy, we find that the arrangements of source azimuth and IID selectivity have significant topography at the scale of single binaural clusters in all eight bats studied. This analysis demonstrates the feasibility of objective quantification of topography and detection of statistically significant topography in experimentally characterized neural maps.

## Methods

**Figure 2:**
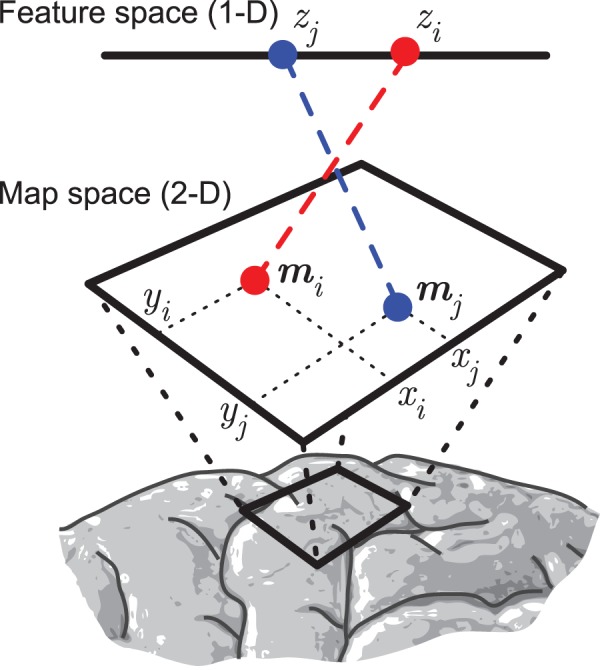
Elements of a topographic map. Fundamental elements of a map from a 1-dimensional feature space to a 2-dimensional map space. Dashed lines represent the link between the positions of neurons in feature space with their positions in map space. Here two neurons 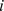 and 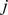 are shown together with their map space (anatomical) and feature space (characteristic stimulus) coordinates.

Before discussing map measures it is useful to establish a formal definition of a map and define some terminology. Here the word ‘map’ refers only to the arrangement of neuronal properties in physical space; no topography is implied. In order to observe a map, it is necessary to identify a number of anatomical elements, the nature of which depends on the experimental technique used. These can be individual neurons in the case of single-unit electrophysiology or multiphoton calcium imaging, local neuronal populations in the case of multi-unit recordings, or local haemodynamic response in the case of fMRI or intrinsic imaging. The units can be arranged in a regular grid (e.g. fMRI voxels) or scattered (e.g. single neurons). The positions of the units in map space (i.e. within the brain) are measured. Often these positions are 2-dimensional, as in cortical maps, but 3-D positions could also be used. Each unit is assigned a label based on its functional properties; typical examples are the characteristic frequency of auditory neurons or the preferred orientation of visual neurons. We refer to the space that the labels are defined within as feature space. Both characteristic frequency and orientation feature spaces are 1-D, but 2-D (e.g. visual neuron receptive field centers) or higher dimensional spaces are possible. For concreteness, all the examples in this article involve a 2-D map space and 1-D feature space (see Figure 2).

### Map measures

A variety of map measures have been used to assess iterative models of topographic map development (see reviews: [11], [23], [24], [27]). Many of these can be directly applied to experimentally measured maps, but some have inherent limitations that prevent this. Some map measures rely on the existence of a known training data set from which the map is derived or learned (e.g. the measure proposed by [28], and the topographic function of [29]), some assume that neurons lie on a regular grid, and some are only applicable where the feature space has the same dimensionality as the map (the directional product measure [24] relies upon the latter two assumptions). For the purposes of this article, we have selected seven measures that can be calculated based on receptive field data alone, and that are flexible with regard to the dimensionality of the feature space and the map space.

When defining a map, we assume that there are 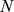 units (neurons), the coordinates of the 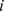
th unit in map space (e.g. on the cortical sheet) are denoted 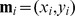, and the position in the one-dimensional feature space (e.g. the preferred stimulus) is denoted 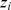. Bold type in equations indicates vector quantities.

Goodhill and Sejnowski [23] described a mathematical framework that unifies a number of different measures of topography. The basis of this framework is the generic measure 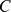:
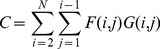
(1)where 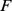 is a distance function in feature space and 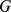 is a distance function in map space. This form, the product of two corresponding pairwise distances summed over all possible pairings of neurons, is the basis for most of the measures described in this article.

#### Pearson distance correlation (PC)

The simplest variants of the 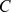 measure are based on Euclidean distances in both feature and map spaces, in this case:
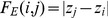
(2)

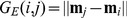
(3)


It is useful to normalize the measure so that maps of different scales or with differing numbers of cells can be compared directly. This can be achieved by computing the Pearson correlation between pairwise distances in feature space and map space (Equation 4). This measure was mentioned by Bezdek and Pal [30], but no results were reported. This measure is also related to the sample distance correlation proposed by Szekely [31], but the latter measure uses centered distances (see reference for details). The Pearson distance correlation is given by:
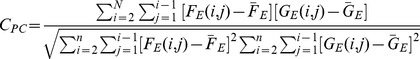
(4)Where 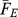 and 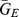 are the mean pairwise distances, for example:



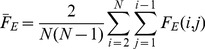
(5)With the Pearson correlation measure it is possible to combine data from different individuals as long as the scale of the map is consistent, as is often the case with subjects of the same age and species. With combined data, it doesn't make sense to compute distances in map space between cells from different subjects, as the coordinate systems may not be aligned and the map shape or orientation may be different. In this case we redefine 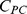 as:
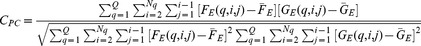
(6)Where 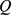 is the number of subjects, 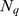 is the number of neurons in the 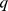
th subject and the mean distances are also computed across all pairs in all subjects. The revised distance functions are:



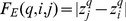
(7)

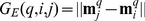
(8)


Here 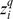 and 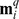 denote the positions of the 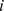
th neuron from the 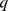
th subject.

#### Spearman distance correlation (SC)

As an alternative to the Pearson correlation, Bezdek and Pal [30] used Spearman's rank correlation coefficient. This is sensitive to the ordering of data and not their absolute values, which means that the measure we denote as 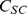 quantifies topology preservation and is not sensitive to distortion of the map unless it results in reordering of the neurons relative to their ordering in feature space. The Spearman distance correlation is given by:
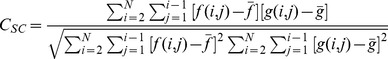
(9)Where 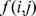 and 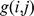 are the tie-corrected ranks of 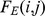 and 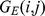 respectively, and 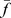, 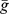 are the mean ranks.

#### Topological correlation (TC)

The topological correlation [32] is another closely related measure, but is based on graph theoretic rather than Euclidean distances. This makes it similar to 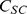 in that it measures similarity of ordering rather than absolute position. To calculate the distances, it is necessary to construct Delaunay triangulations (see e.g. [33]) in both map and feature spaces. The geodesic distance in map space 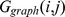 between units 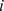 and 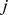 is the number of edges in the shortest path connecting them in the Delaunay triangulation. For the 1-D feature space the Delaunay triangulation is undefined, so rank difference is used instead:
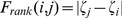
(10)Where 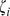 is the tie-corrected rank of 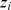. The topological correlation 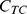 is then defined as:



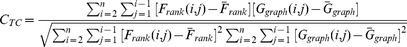
(11)Again, 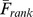 and 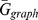 are the mean distances over all pairs of cells.

#### Wiring length (WL)

The minimum wiring measure (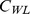) is designed to estimate the length of axonal ‘wiring’ required to connect all pairs of cells that are neighbors in feature space (e.g. are selective for neighboring stimuli). This measure is a normalized version of the ‘minimum wiring’ objective function used by Goodhill and Sejnowski [23]. In this case the distance functions are defined as:
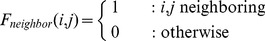
(12)

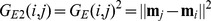
(13)


Neighboring units are defined as those with identical or adjacent positions in feature space. 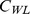 is then defined as:
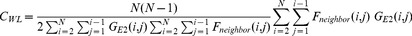
(14)


#### Path length (PL)

The path length measure 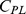 is the same as wiring length, but the roles of map and feature spaces are reversed. The distance measures are:
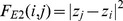
(15)

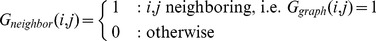
(16)


For the purposes of this article, we define neighboring in terms of the Delaunay triangulation as the neurons are not located on a regular grid as was the case when this measure was investigated by Goodhill and Sejnowski [23]. As with the wiring length, the path length measure is normalized to make it independent of map size or measurement units. 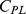 is defined as:
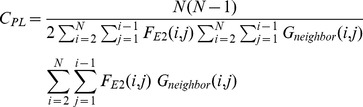
(17)


#### Zrehen measure (ZM)

This measure quantifies local consistency in maps and is a normalized version of the measure proposed by Zrehen [34]. It measures the separation in feature space of neurons that are neighbors in map space. Although originally applied to model neurons arranged in a regular grid, here we use the Delaunay triangulation to determine which neurons are neighbors. The distance measures used are 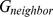 (Equation 16) and a modified version of 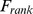 that counts the number of interposing ‘intruders’ in feature space between the neighboring neurons:
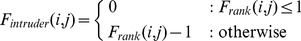
(18)


The measure 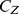 is then defined as:
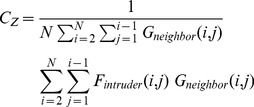
(19)


#### Topographic product (TP)

Bauer and Pawelzik's topographic product [35] is the only measure examined in this article that does not fit into the 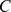 framework of Goodhill and Sejnowski (Equation 1). The topographic product 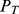 is a measure of the preservation of neighbor relations based on Euclidean distances. Bauer and Pawelzik first defined 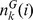 as the index of the 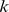
th nearest neighbor of neuron 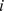, in terms of distance in map space 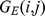 (Equation 3), and 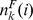 as the 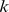
th nearest neighbor of cell 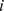 in feature space (i.e. in terms of 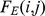, Equation 2). They then defined the ratios:
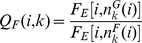
(20)

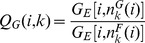
(21)


The geometric mean over all neighbors within a given neighborhood size 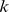 is given by:
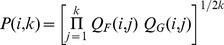
(22)


If a map is perfectly ordered and all neighborhood relations are preserved, then 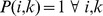. The topographic product 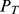 is a measure of the deviation of 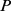 from 1 (by taking logarithms), averaged over all neurons and all possible neighborhood sizes (Equation 23). Our definition of 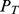 differs slightly from that of Bauer and Pawelzik in that we take the absolute value of 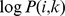 before averaging; this makes 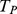 non-negative and more suitable for use in a permutation test.
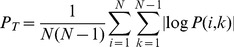
(23)


A problem arises when two or more neurons have identical positions in either feature or map space, as this means that the order of neighbors is not always well defined. To resolve this issue, we use a Monte Carlo (MC) approach: the final value of 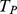 is taken to be the mean of 1000 samples in each of which the order of equidistant neighbors is randomly permuted.

### Significance testing

For all of the measures described above, Monte Carlo permutation tests were used to calculate 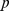-values. Taking the generic 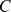 measure as an example, the 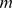
th of 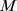 Monte Carlo samples is given by:
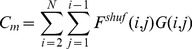
(24)

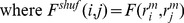
(25)Where 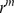 is the 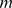
th randomly permuted instance of a vector containing the integers 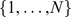. In other words, for each sample the feature space positions were randomly shuffled and the measure computed using the shuffled values. We then compute 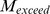, the number of samples that are more ordered than the actual map:



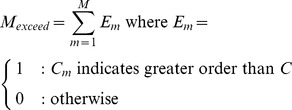
(26)The 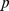-value is given by:
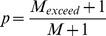
(27)


All results in this article are based on MC sample sizes of 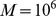 unless it was faster to perform an exact permutation analysis (i.e. where 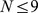). To control for multiple tests, the Benjamini-Hochberg step-up procedure [36] was used to obtain corrected 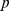-values. To test significance of the multiple-subject 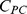 measure, the method above was modified so that feature space data were pooled across all subjects before shuffling.

### Map models

**Figure 3:**
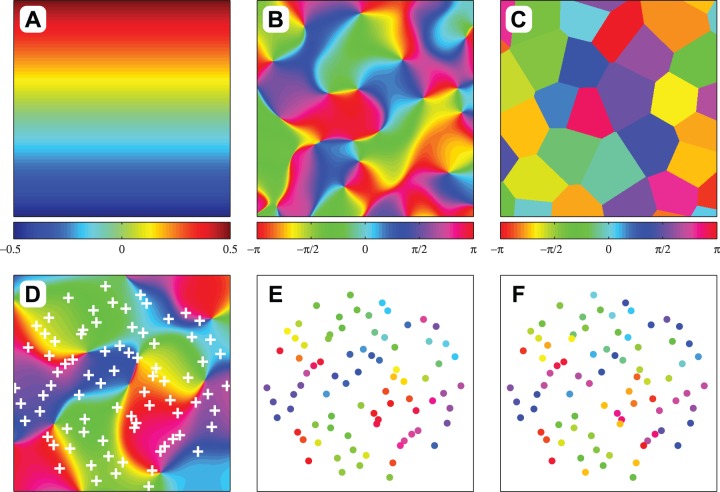
Map models and spatial sampling. Three map models were used to investigate the sensitivity of map measures to different forms of topography: (A) linear map, (B) angle map and (C) clusters (latter two with scale parameter 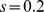). The sampling process is illustrated in the lower three panels: (D) raw angle map (scale parameter 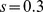) with quasi-random sample locations marked (number of points 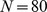), and sampled ‘neurons’ before (E) and after (F) noise was added (SNR  = 3).

To assess the sensitivity of the measures to different forms of topography, three generative map models were used (see Figure 3A–C). The map models were used to generate 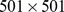 arrays defining the ground truth tuning properties. This array was then sampled at 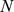 quasi-random points and noise was added to the samples. The map measures were then used to quantify the order in the noisy samples.

#### Linear map

The simplest map was a linear gradient intended to model maps with smooth large-scale structure (see Figure 3A). The linear map 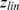 was defined as:
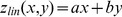
(28)Where 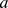 and 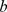 are drawn from a uniform distribution on the interval 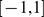 and 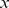, 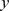 are both in the interval 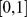.

#### Angle map

To represent maps with a convoluted structure, such as visual cortex orientation maps (see Figure 3B), we used random angle maps derived from bandpass filtered white noise [37], [38]. These maps are synthesized by generating 2-D arrays 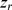 and 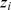 of Gaussian white noise and convolving them with a ‘mexican hat’ bandpass filter kernel (Equation 29). Treating the two arrays of filtered noise as the real and imaginary parts of an array of complex numbers, the angle map is found by taking the argument (Equation 30).
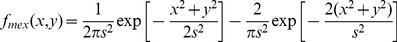
(29)

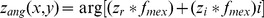
(30)


The filter scale parameter 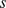 determines the characteristic size of the aperiodic map features. As the feature space of this type of map is periodic, circular distance metrics and circular statistics [39] were used when computing all map measures of angle and cluster maps.

#### Clustered arrangement

This model was designed to test the sensitivity of measures to local consistency where there was no larger-scale topography. Clusters were generated by drawing 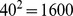 quasi-random seed points from a Halton sequence ([40], Matlab implementation), and generating a Voronoi tessellation from these points. The seed point coordinates and tessellation were then rescaled by a factor of 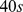; this yields approximately equivalent scaling of angle and cluster maps for any given value of the scale parameter 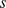. The 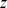 values for each seed point were then drawn from a uniform distribution and the pixel 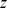 values set to the 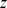 value of the nearest seed point, thus ‘coloring’ the Voronoi tessellation. Random variation of the tessellation was achieved by randomly setting the skip parameter (number of initial points in the Halton sequence to be discarded) when calling Matlab's haltonset() function.

#### Spatial sampling procedure

The process of measuring a biological map was modeled by quasi-random sampling of the maps and the addition of noise (see Figure 3D–F). The locations of the observation points were again drawn from a Halton sequence with a randomly chosen skip value. Points outside the unit disc were rejected to ensure that measure values were independent of map orientation. To simulate random neuronal variability and measurement error, Gaussian noise was added to the 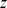 values of each sample. The variance of the noise was defined in terms of the signal to noise ratio (SNR). For periodic feature spaces, the variance was computed using the CircStat toolbox [39].
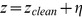
(31)

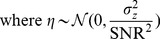
(32)Where 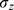 is the standard deviation of the feature space coordinate 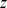 across the whole map.

### Pallid bat auditory cortical maps

The pallid bat echolocates for general orientation and obstacle avoidance and listens to prey-generated noise to localize and hunt terrestrial insects [41]. A1 in the pallid bat consists of two subregions, one that is specialized for the processing of frequency modulated echolocation calls and a second that responds to broadband, noise-like sounds. This second region is likely to be important for passive detection and localization of prey [26]. The passive hearing subregion is further divided into at least two clusters of neurons based on IID selectivity: the ‘peaked’ cluster and the binaural inhibition (EI) cluster (following the nomenclature of [25]). The peaked cluster is made up of neurons that respond to sounds arriving with similar amplitude at both ears, and have bell-shaped azimuth tuning functions, while the EI cluster consists of neurons that are excited by input from the contralateral ear and inhibited by the ipsilateral ear, which leads to sigmoidal azimuth tuning functions.

**Figure 4:**
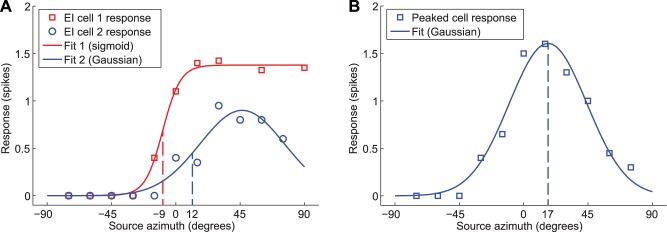
Tuning functions and characteristic stimuli. Examples of typical tuning functions of (A) EI cells and (B) peaked cells in pallid bat primary auditory cortex. Parametric tuning functions (solid lines) were fitted to the measured responses. EI neurons were assigned characteristic azimuth labels (indicated by dashed lines) where the fitted tuning function was equal to 50% of the maximum response. For Peaked neurons, the characteristic azimuth was defined as the peak of the fitted tuning function. IID tuning functions and characteristic stimuli were determined similarly.

All data were collected as described by Razak [25]. In this article we analyze source azimuth, IID and frequency selectivity mapping data from four bats (corresponding to the maps shown in figures 4–6 of [25]) together with source azimuth and frequency selectivity data from a further four bats. For the tonotopic maps, characteristic frequencies were determined as described by Razak [25].

**Figure 5:**
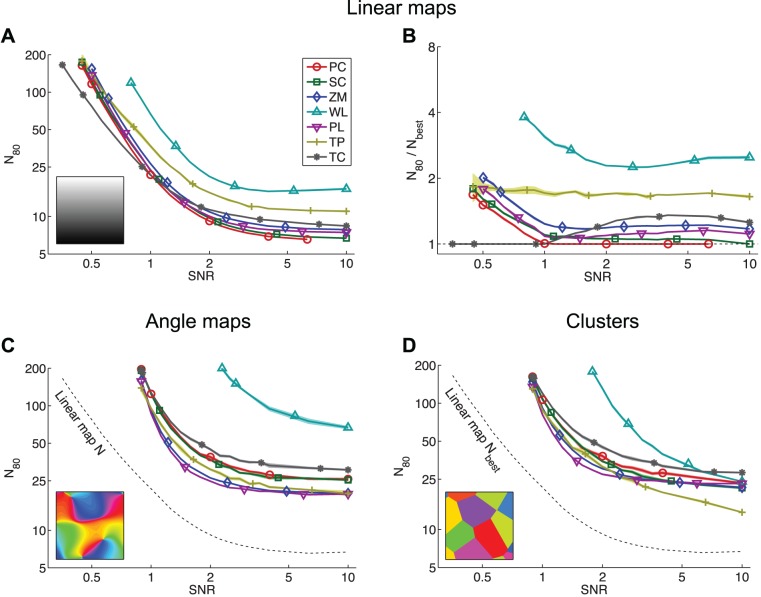
Comparison of the statistical power of seven map measures (PC: Pearson distance correlation, SC: Spearman distance correlation, ZM: Zrehen measure, WL: wiring length, PL: path length, TP: topographic product, TC: topological correlation) when detecting (A) linear maps, (C) angle maps and (D) clusters. Power is summarized by the quantity 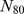, the mean number of points (e.g. neurons, voxels) required to achieve a statistical power of 80%; this is shown as a function of the SNR. Panel B shows the relative powers of the measures for linear map detection; here 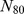 is normalized by 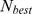, the 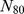 of the most powerful measure for a given map type and SNR. For the angle maps and clusters the scale parameter 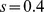 and the insets show examples of the corresponding map type and scale. All axes have logarithmic scales. Missing data indicate that 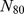 is outside the range 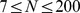. Uncertainty is depicted by shaded regions of 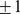 StdErr.

**Figure 6:**
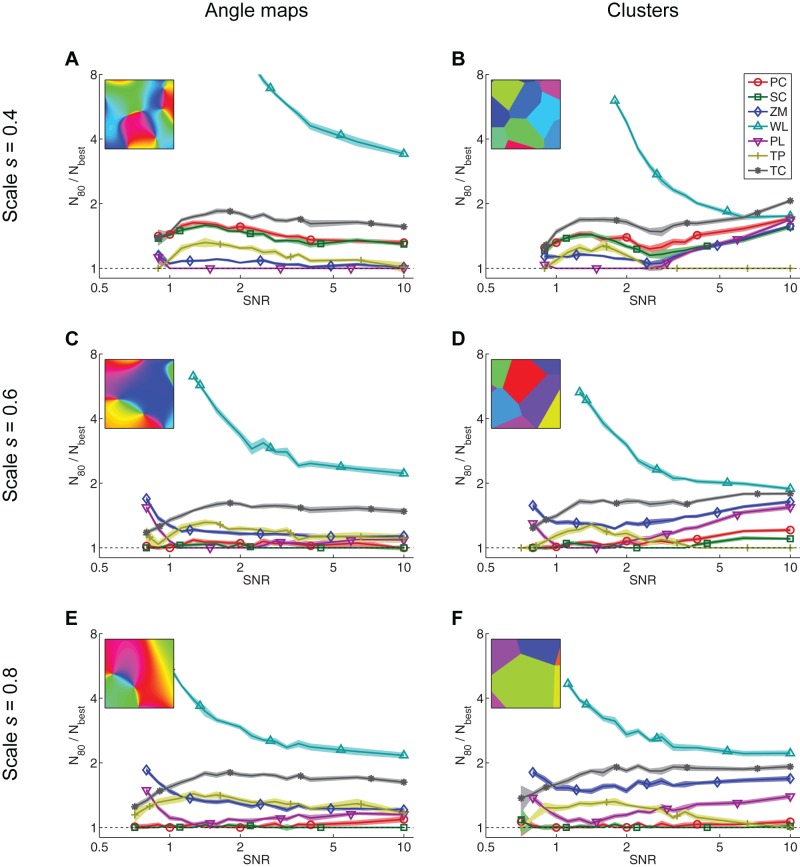
Relative power of measures for detecting maps of various scales and types. For each map measure, the plots show the number of data needed for reliable detection of angle maps (A, C, E) and clusters (B, D, F). To show the relative power more clearly, 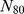 is normalized by 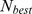, the 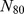 of the most powerful measure for a given map type and SNR. The more powerful the measure, the lower it appears on the plots. It can be seen that the map type i.e. angle map vs. clusters, has little effect upon the relative powers of the measures; the ordering of the measures in terms of power is similar for both forms of map. All axes have logarithmic scales. Missing data indicate that 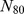 is outside the range 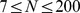. Uncertainty is depicted by shaded regions of 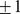 StdErr.

#### Azimuth labeling

To allow interpolation between the 15° azimuth spacing of the raw data, parametric tuning functions were fitted to the data (Figure 4). For each EI cell, Gaussian (Equation 33) and sigmoidal (Equation 34) curves were fitted; further analyses were based on the better fitting of the two. Peaked cells were fitted with Gaussian tuning functions (Equation 33). The functions are defined as:
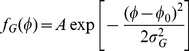
(33)

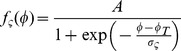
(34)Where 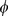 is the source azimuth, 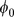 and 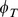 are preferred azimuth (azimuth eliciting maximum response) and transition azimuth (azimuth of maximum gradient) respectively, and 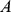 defines the maximum response (normalized spike count). The width parameters 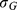 and 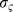 define the azimuth range over which the neuron responds in the Gaussian case, and the slope of the transition in the sigmoidal case respectively. Minimum values (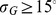, 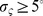) were imposed on the width parameters to avoid over fitting.

For peaked cells, the azimuth label is simply the azimuth eliciting maximum response 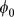. For EI cells, the azimuth label 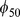 was defined as the ipsilateral (up-crossing) point where the tuning function is equal to 50% of its maximum. For sigmoidal tuning functions this is simply 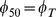. For EI cells with Gaussian fitted tuning functions 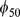 is given by:
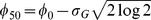
(35)


#### IID labeling

IID data was treated in a similar way to the source azimuth data. In this case the parametric tuning functions are:
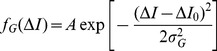
(36)

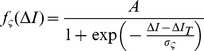
(37)Where 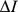 is the IID, and 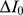 and 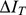 are the IIDs associated with maximum response (Gaussian) and maximum tuning function gradient (sigmoidal). The width parameters were constrained (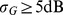, 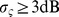) to avoid over fitting. Again, the feature space labels for EI cells were defined as 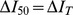 for sigmoidal and 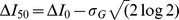 for Gaussian tuning functions, and the labels for peaked cells were defined as 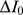, the IID corresponding to the maximum response.

## Results

### Detection power of map measures

We assessed the sensitivity of seven map measures by using them to quantify the topography in artificial ‘electrophysiological’ (i.e. spatially scattered) mapping data. The measures are: Pearson distance correlation (PC), Spearman distance correlation (SC), Zrehen measure (ZM), wiring length (WL), path length (PL), topographic product (TP) and topological correlation (TC); see Methods for definitions. Mapping data was generated by sampling an underlying map at spatially scattered locations, then adding random noise to the feature space coordinates of the samples (see Figure 3D–F). By varying the signal-to-noise ratio (SNR) and the number of points at which the map was sampled, we examined the relationship between the strength of the map (in terms of SNR) and the number of points needed for reliable topography detection using each measure. We defined reliable detection as a statistical power of 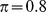 at a significance level of 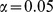 i.e. an 80% chance of correct detection, and the number of points needed to achieve this is denoted as 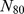. 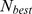 is the 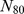 of the most powerful measure for a given SNR and map type. Additional simulations (results not shown) showed that the findings described in this article are robust with respect to small changes in either significance level 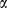 or the detection threshold statistical power 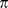. Clearly, increasing the sample size increases the statistical power of any test and increasing the density of measurement points increases the ability to detect patterns at smaller scales. Here we focus on comparing the statistical powers of the map measures to identify which measures are most powerful and hence are likely to be most useful for detecting topographic organization in experimental datasets.

Figure 5A shows the relationship between SNR and the number of points 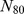 required for reliable detection when the underlying map is a simple linear gradient (as in Figure 3A). The more powerful a measure is, the lower its line appears on the plot; the most powerful measure at any given SNR is that which achieves reliable detection with the least data and hence is the lowest line on the plot. For linear maps, the topological correlation (TC) is the most powerful measure for detecting maps with weak topography (that is, maps heavily corrupted with noise: SNR 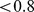), while the Pearson distance correlation (PC) is the most powerful measure for maps with strong topography (SNR 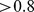). Four of the measures (PC, SC, ZM and PL) have similar power across the SNR range. WL and TP are consistently less powerful, requiring 1.6 to 4 times as many data as the most powerful measure at any given SNR to achieve the same statistical power (Figure 5B).

**Figure 7:**
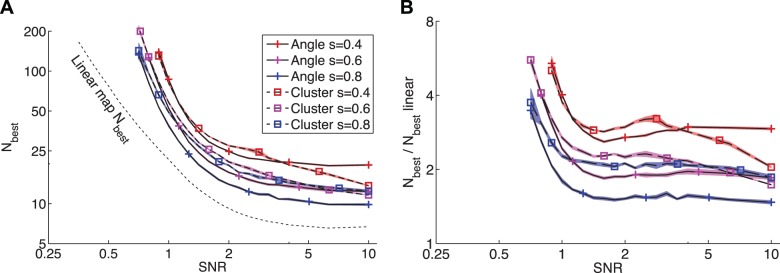
The effect of map scale on nonlinear map detection. Larger scale maps can be detected with fewer data. Panel A shows 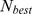 (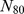 of the most powerful measure) for angle maps and clusters at three different scales: 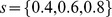. Panel B shows the detectability of each type and scale of nonlinear map relative to a linear map with the same SNR i.e. 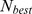 normalized by 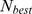 for a linear map. All axes have logarithmic scales. Missing data indicate that 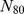 is outside the range 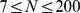. Uncertainty is depicted by shaded regions of 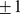 StdErr.

Neural maps generally have structures more complex than a linear gradient. We next assessed the statistical power of the same seven measures for detecting two forms of nonlinear map: convoluted angle maps similar to V1 orientation maps (Figure 5C), and clustered arrangements where there is no overall topography, but tuning properties are locally homogeneous (Figure 5D). The nonlinear maps are only locally consistent, so higher sampling densities are required in order to detect the map. When used for the detection of these nonlinear maps, the statistical power of the measures depends primarily upon the spatial scale of the map, as well as the SNR and number of points; the effect of map form (angle map versus clusters) is relatively minor (Figure 6). The parameter 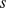 controls the spatial scale of the model maps and hence the density of measurements required to resolve the map. Maps with larger features (greater 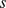) can be detected with fewer measurements than smaller-scale maps, as can be seen in Figure 7. The most powerful measures for detecting smaller-scale nonlinear maps (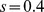) are the path length and topographic product (Figures 6A and 6B), despite the fact that the topographic product is one of the least powerful measures for detecting linear maps (see Figure 5B). For detecting both angle maps and clusters at larger scales (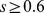), the most powerful measures are the Pearson and Spearman distance correlations, path length, and the topographic product for cluster maps at very high SNR (Figure 6C–F). The Zrehen measure also has relatively high power for detecting nonlinear maps, particularly at smaller scales, but it is never the most powerful measure.

To summarize, the relative power of the measures varies according to both the type (mainly linear versus nonlinear) and scale of the map, as well as the SNR. The conventional correlative measures (Pearson and Spearman distance correlations) are the most powerful for detecting large scale topography i.e. linear maps and larger-scale nonlinear maps (approximately 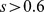). An angle map on an infinitely large scale is equivalent to a linear map, so it is not surprising that the same measures are most effective at detecting linear and large-scale nonlinear maps. The path length and topographic product are the most powerful for detecting the localized topography in smaller-scale nonlinear maps (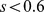).

### Pallid bat A1 maps

To illustrate how statistical tests can be used to objectively determine the existence of neural topographic maps, we quantified the topography of three different neuronal tuning properties in pallid bat A1. Using data gathered from 211 cells in the EI and peaked clusters of eight bats (see Methods and [25]), we tested for the existence of significant maps of frequency (tonotopy), IID and source azimuth. IID and azimuth labels corresponding to steeply sloping regions of the tuning curves, rather than maxima, were chosen because cells in the EI cluster generally have sigmoid-like tuning curves without clearly defined maxima. This means that the IID and azimuth maps in the EI cluster differ from typical place maps (as in e.g. the superior colliculus) where the locus of activity directly reflects the value of the stimulus variable. In the peaked cluster, the azimuth labels were located at the peaks of the tuning curves. For the purposes of this article, we have used the term ‘tuning curve’ to refer to any function that relates an arbitrary stimulus to a response firing rate; it does not imply sound frequency selectivity in particular. The pallid bat data was analyzed directly; there was no subsampling or other preprocessing of the data aside from determining the characteristic stimulus labels (see Methods).

Significant topographic maps of characteristic frequency were detected in all 8 bats (Table 1). In all 8 bats significant tonotopy was also detected when the EI clusters were considered in isolation. Tonotopy was also significant within the peaked cluster in 3 of 4 bats for which data from the peaked cluster was available. These results are consistent with the tonotopic arrangement of auditory cortex found in many species.

**Table 1:** Proportion of bats with significant tonotopic, IID and azimuth maps.

	Frequency map			IID map		Azimuth map		
Measure	EI	Peaked	EI+Peaked	EI	Peaked	EI	Peaked	All cell groups
PC	5/8	2/4	3/4	2/4	0/4	5/8	1/4	52.5%
SC	6/8	2/4	4/4	2/4	0/4	5/8	0/4	55.0%
ZM	5/8	2/4	4/4	3/4	0/4	7/8	1/4	62.5%
WL	6/8	2/4	3/4	1/4	0/4	2/8	0/4	37.5%
PL	6/8	2/4	4/4	3/4	0/4	6/8	3/4	70.0%
TP	8/8	3/4	4/4	3/4	0/4	4/8	0/4	62.5%
TC	5/8	2/4	4/4	3/4	0/4	5/8	0/4	57.5%
Any measure	8/8	3/4	4/4	3/4	0/4	8/8	3/4	

Results of map detection analysis of pallid bat data. Each table cells shows the number of bats in which significant maps were detected/total number of bats from which data were available. The ‘any measure’ row shows the number of bats where significant topography was detected by at least one measure. Each column relates to a given tuning property (e.g. frequency) and group of neurons (e.g. cells from the EI cluster). The ‘all cell groups’ column gives combined detection rates for each measure across all maps in all animals; this is a coarse indication of the relative power of the measures.

Systematic cortical maps of IID and source azimuth selectivity are present within the EI cluster in the pallid bat [25], [42]. Our results confirm the presence of a systematic arrangement of IID and azimuth tuning within the EI cluster. Significant topography in IID maps was detected in the EI cluster in 3 of 4 bats for which IID data were available, but no significant IID topography was found in the peaked cluster (Table 1). Significant azimuth maps in EI were detected in all eight bats, but in the peaked cluster topography was much weaker (as in [25]), being marginally significant (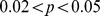 after Benjamini-Hochberg correction) in 3 of 4 animals from which data were available (Table 1).

The Pearson distance correlation measure allows us to combine data from multiple animals into a single statistic to assess the strength of topography across the population (see Methods). This population analysis provides additional confirmation of highly significant tonotopy and highly significant topographic arrangement of IID and azimuth selectivity (see Table 2). Although the azimuth map is only marginally significant in 3 of 4 animals for which peaked cluster data is available, it is clearly significant (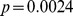) when the data from the four bats are combined. This analysis is also useful because the Benjamini-Hochberg procedure used to correct for multiple tests in the individual analysis is not very conservative and can, at best, be expected to give a false discovery rate of 0.05, equivalent to approximately 8 tests wrongly identified as significant. Combining the data into a single statistical test, or a much smaller number of tests, avoids the difficulties associated with correcting for large numbers of tests.

**Table 2:** Map detection analysis of combined map data from all 8 bats.

	Frequency map			IID map		Azimuth map	
	EI	Peaked	EI+Peaked	EI	Peaked	EI	Peaked
*C_PC_*	0.27	0.52	0.34	0.25	−0.019	0.30	0.33
*p*	<10^−4^	<10^−4^	<10^−4^	0.0017	3.9	<10^−4^	0.0024
*n*	156	49	205	71	42	156	49

Results of the analysis of combined data from all animals. Columns indicate the tuning property (e.g. characteristic frequency) and cell class (e.g. EI). Pearson distance correlation 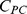, 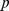-value (Bonferroni corrected, 7 tests) and number of neurons 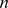 are given for each candidate map i.e. combination of tuning property and cell class. The Bonferroni method of correcting for multiple tests can lead to corrected 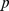-values greater than 1, as is the case for IID tuning in the peaked cluster.

One possible explanation for the systematic arrangements of IID and azimuth selectivity is that they are somehow a consequence of tonotopy. To test this hypothesis we calculated the correlation between characteristic frequency and 50% IID, and between characteristic frequency and 50% azimuth. In the EI cluster, only one significant correlation was found, between characteristic frequency and azimuth in bat PAL28 (Pearson 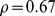, 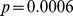). There was no significant correlation between characteristic frequency and azimuth in the EI cluster when data from all 8 animals was combined, or between IID and characteristic frequency in any bat or across all bats. In the peaked cluster there was no significant correlation between azimuth and characteristic frequency. There is, however, a significant negative correlation between IID and characteristic frequency in the peaked cluster at the population level (Pearson 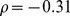, 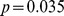). Interestingly, this correlation does not result in the significant tonotopy also manifesting as a significant IID map (see Table 2). In summary, the systematic arrangements of azimuth and IID selectivity do not appear to be a consequence of tonotopy.

It is useful to compare the pallid bat data to the map models discussed in the previous section. Both the linear and cluster map models are plausible candidates for the underlying form of the pallid bat azimuth and IID maps (tonotopy is locally and approximately linear). If it is assumed that only frontal space is represented, or that frontal space predominates, then both the azimuth and IID feature spaces are non-periodic and the space maps could be linear (perhaps oriented near-perpendicular to the tonotopic gradient). Alternatively, the space map could take the form of clusters as this is a known organizational principle of A1 (see e.g. [43]). One approach to resolving this question is to fit the models to the experimental data. Both the cluster and angle map models are under-constrained by the data; for any possible set of mapping data, there are an infinite number of possible angle or cluster maps that would explain the data perfectly. Fitting the linear model, however, was straightforward and allowed us to estimate the SNR of the bat data based on the assumption of an underlying linear map. To do this, we fitted a bilinear function that predicts the selectivity feature 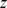 for a given location on the cortex defined by 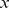 and 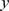. The SNR of the data was then estimated by calculating the proportion of the standard deviation of 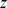 that was explained by the bilinear fit. The best frequency maps had estimated SNRs between (approximately) 0.9 and 2, while the IID and azimuths maps had estimated SNRs between 0.4 and 2. The estimated SNR indicates how well the pallid bat mapping data is explained by a linear model, and the broad range of observed SNRs suggests that the linearity of the maps varies considerably between animals. While the results of the permutation tests show that azimuth and IID tuning is organized non-randomly, it is not possible to say conclusively what form the azimuth and IID maps take; this question can only be addressed by further mapping using a technique with higher spatial resolution.

## Discussion

We have shown that topography in the anatomical layout of neuronal tuning properties can be quantified using measures that do not rely on any prior knowledge about the form of the map. These measures can be used to perform statistical tests for the existence of significant topography. This provides an objective method for detecting topographic maps that are unclear, for instance where data are available from only a small number of neurons, or the scale of map features are close to the spatial resolution of the measurement technique. A Matlab toolbox containing implementations of all measures and statistical tests described in this article is available for download from GitHub (https://github.com/StuYarrow/MapTools).

We assessed the sensitivity of seven measures (Pearson distance correlation, Spearman distance correlation, Zrehen measure, wiring length, path length, topographic product and topological correlation; see Methods for definitions) to linear and nonlinear model maps obscured by adding noise to the characteristic stimulus values. Sensitivity was quantified by calculating the statistical power, for map detection, of permutation tests based on each measure. The sensitivity of measures depended on the form (linear vs nonlinear) and scale of smoothness in the map, and on the SNR of the characteristic stimulus labels; no one measure was the best at detecting all maps. For detecting linear maps the Pearson and Spearman distance correlations and the topological correlation were the most powerful. For larger-scale nonlinear maps the Pearson and Spearman distance correlations are among the most powerful, while the path length and topographic product are more powerful at detecting smaller-scale nonlinear maps.

One of the criteria used to select the measures was that they should be flexible in terms of the dimensionality of feature and map spaces. It is therefore a limitation of this study that only 1-D feature spaces and 2-D map spaces were addressed. The dimensionality of feature spaces in particular can vary greatly depending on how the stimulus space is decomposed into features, and how many of these features are taken into account in an analysis. Another limitation was the way that we modeled degradation of the map by adding random noise to the feature space positions. In reality, natural variability of maps is likely to be much more complex and could involve processes very different from independent random noise, for example warping or fracturing of the map.

Although we have focussed on the use of map measures in statistical tests for map detection, the same measures have other potential applications. They could be used, for example, to assess differences in map orderliness between anatomical regions, developmental stages or experimental groups, or changes in maps as a result of aging or changes in properties of the environment.

Our results confirm the presence of systematic arrangements of spatial (azimuth) and binaural (IID) selectivity in pallid bat A1. It has been suggested previously that topographic arrangements of IID and azimuth selectivity exist within the EI cluster [25], [26], [42], however these maps were identified only subjectively, and the strength or clarity of the maps varies considerably between animals. Razak showed that the overall level of activation of the EI cluster varied systematically with source azimuth (Figure 7 of [25]), but did not give any quantitative evidence for a systematic relationship between tuning properties and locations of neurons. The identification of systematic arrangements of IID and source azimuth selectivity in the pallid bat raises the question as to whether similar maps are also present in other species.

It is important to note that the systematic arrangements of IID and azimuth selectivity in the pallid bat are confined to clusters of neurons with similar patterns of binaural selectivity. Binaural clusters are a ubiquitous organizational feature of the auditory cortex across species. It remains unclear if systematic maps of source azimuth are present in the intrinsic organization of binaural clusters in other species because such studies have not yet been conducted. In the pallid bat these binaural clusters are of the order of 1 mm across, so measuring maps within the clusters requires a technique with spatial resolution of the order of 0.1 mm. In addition, the focus needs to be on mapping within binaural clusters across isofrequency contours as most previous studies have concentrated on mapping along isofrequency contours, potentially spanning multiple binaural clusters. The characterization of internal organization of binaural clusters in other species will have significant consequences for our understanding of how auditory spatial information is represented in the cortex and is an important area for future research, particularly given the availability of high-resolution techniques such as multiphoton calcium imaging and multi-electrode arrays.

One feature of the pallid bat data that is visible in our results is the variability in the apparent orderliness of the maps between animals; in some bats highly significant topography is detected by many measures, but in a few cases the map measures show only weak topography. The results of the population analysis (Table 2) show that there is significant topography in azimuth tuning in the the EI (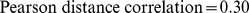, 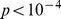, 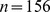) and peaked (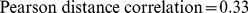, 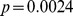, 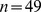) clusters, and in IID tuning in the EI cluster (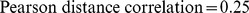, 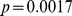, 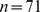) when the data from all bats is considered together. There are a number of possible reasons for the observed differences between bats: random sampling variability, measurement error in recording and extracting tuning curves and characteristic stimuli, individual differences in the strength of the map or in the form of the map (e.g. warped or fractured maps).

For the purposes of this article, we have defined a topographic map as any systematic relationship between a given tuning property and the physical location of a neuron. This is perhaps a broader definition than is typically used, because it is not limited to place maps where the characteristic stimuli – the positions of the neurons in feature space – correspond to firing rate maxima. In the pallid bat, the arrangements of azimuth and IID tuning in the EI cluster are examples of maps that are not ‘place’ maps; in these cases the tuning curves have no well-defined peak and the characteristic stimulus labels on the slopes of the tuning curves are used. The question of what stimulus value to use to characterize or represent a neuron is a nuanced one. Traditionally, those that elicit the maximum response (i.e. the location of the tuning curve peak) have been used, but this doesn't make sense when the tuning curve is monotonic and has no distinct maximum. One approach would be to use the stimulus value that the neuron conveys the most information about, but this is not easy to determine; maximum information depends on a number of factors and can coincide with steeply sloping regions of the tuning curve, or the peak, or somewhere in between [44], [45]. Using one characteristic stimulus to represent the entire receptive field is clearly a simplification, albeit one that is widely accepted. If this simplification was to prove problematic in the future, it would be possible to adopt new map measures using the same basic form given in Equation 1, but using distances in the higher-dimensional space of tuning functions rather than distances between one-dimensional characteristic stimuli.

The measures used in our analysis were chosen for their flexibility and can be applied to a wide variety of datasets with different dimensionalities of map and feature spaces. Here we have only addressed one-dimensional feature spaces and two-dimensional map spaces, so further work is required to investigate the properties of the measures in spaces with other dimensionality. Feature spaces with more than one dimension will be of particular interest. These methods could also be applied to grid-like mapping data, for example fMRI data. With this type of data, the regular spatial sampling and greater number of measurements may affect the relative statistical power of the measures, so further investigation of the properties of these map measures using simulated gridded data would be valuable.

### Conclusion and recommendations

Topography in neural maps can be objectively quantified using measures that compare the pairwise anatomical (map space) distances between neurons with the pairwise distances in some feature space, for example the difference in preferred stimulus. Correlation between these two distances indicates a tendency toward topographic arrangement of the feature. By applying a permutation test, these measures can be used to determine whether a suspected neural topographic map is statistically significant; this is valuable where the topography is weak or unclear, the measurements are noisy, the number of data is limited, or the characteristic scale of map features is close to the spatial resolution of the measurement technique. The way in which map space and feature space distances are quantified determines the type of map that the measure is most sensitive to. Some measures (particularly the Pearson and Spearman distance correlation) are more effective, relative to other measures, at detecting the large-scale smoothness found in linear or larger-scale nonlinear maps than they are at detecting localized topography in smaller-scale nonlinear maps (see Figure 6). The opposite is true for other measures, particularly the topographic product. The wiring length and, to a lesser extent, topological correlation measures had relatively low statistical power for map detection in general.

The approach used to test for the presence of significant topography might be guided by the investigator's prior knowledge about the form of the map. If the map is thought to be linear (e.g. a tonotopic or retinotopic map), or convoluted on a scale where map features are many times larger than the distance between recording sites, either the Pearson or Spearman distance correlation would be a good choice of measure. For nonlinear maps with smaller features, the topographic product or path length are likely to be a good choice. If the form of the map is unknown, more than one measure might be used (e.g. the Pearson or Spearman distance correlation together with the topographic product) and a suitable method used to correct for multiple tests. If the Pearson distance correlation is used, data from multiple subjects can be combined in a single permutation test for detecting topography, without the need for registering or otherwise preprocessing the data. This approach offers the possibility of detecting maps at the population level when the topography is too weak, or insufficient data are available to support detection in individual subjects.

The results of our analysis of pallid bat mapping data confirm that topographic maps of source azimuth and IID exist within binaural clusters in pallid bat primary auditory cortex. As auditory spatial tuning properties within binaural clusters have not been mapped in any other species with sufficient resolution to identify equivalent maps, our findings suggest that high-resolution mapping of spatial tuning properties in the auditory cortex is likely to be important in understanding how auditory space is represented in the cortex.
